# Real-time detection of road manhole covers with a deep learning model

**DOI:** 10.1038/s41598-023-43173-z

**Published:** 2023-09-30

**Authors:** Dangfeng Pang, Zhiwei Guan, Tao Luo, Wei Su, Ruzhen Dou

**Affiliations:** 1 School of Mechanical Engineering, Tianjin Sino-German University of Applied Sciences, Tianjin, 300350 China; 2 School of Automobile and Rail Transportation, Tianjin Sino-German University of Applied Sciences, Tianjin, 300350 China; 3 School of Electronic Information and Automation, Tianjin University of Science and Technology, Tianjin, 300222 China; 4 Tianjin SOTEREA Automotive Technology Co., Ltd., Tianjin, 300308 China

**Keywords:** Electrical and electronic engineering, Mechanical engineering

## Abstract

Road manhole covers are crucial components of urban infrastructure; however, inadequate maintenance or poor marking can pose safety risks to vehicular traffic. This paper presents a method for detecting road manhole covers using a stereo depth camera and the MGB-YOLO model. We curated a robust image dataset and performed image enhancement and annotation. The MGB-YOLO model was developed by optimizing the YOLOv5s network with MobileNet-V3, Global Attention Mechanism (GAM), and BottleneckCSP, striking a balance between detection accuracy and model efficiency. Our method achieved an impressive accuracy of 96.6%, surpassing the performance of Faster RCNN, SSD, YOLOv5s, YOLOv7 and YOLOv8s models with an increased mean average precision (mAP) of 15.6%, 6.9%, 0.7%, 0.5% and 0.5%, respectively. Additionally, we have reduced the model's size and the number of parameters, making it highly suitable for deployment on in-vehicle embedded devices. These results underscore the effectiveness of our approach in detecting road manhole covers, offering valuable insights for vehicle-based manhole cover detection and contributing to the reduction of accidents and enhanced driving comfort.

## Introduction

Road manhole covers are essential components of urban infrastructure, facilitating access to underground utility networks such as sewage, gas, and water systems. However, when these manhole covers are not adequately maintained or marked, they can pose significant safety risks to vehicular traffic. In the realm of traffic safety operations, the swift recognition of road environments by vehicles is crucial as it can significantly enhance driving safety. Recent years have seen many researchers focusing on the use of deep learning methods for identifying road participants such as automobiles, pedestrians, and traffic signs. However, comparatively, there is limited research on the recognition and detection of road manhole covers. If not properly maintained or marked, these covers could pose a safety hazard to vehicular traffic. Thus, there is a pressing need to explore and develop more efficient methods for manhole cover detection and recognition to fill this research gap, which could profoundly impact the safety and efficiency of the entire traffic operation system.

During the implementation of autonomous driving, most of the terminal devices used in vehicles are constrained by environmental factors such as space and power. These devices are predominantly low-power and have limited computational resources. In recent years, deep learning models have shown remarkable capabilities in various computer vision tasks, including object detection. Among these models, the YOLO^1^ (You Only Look Once) architecture has gained significant attention due to its ability to achieve real-time object detection by dividing the image into a grid and predicting the bounding boxes and class probabilities within each grid cell. However, existing YOLO models still face challenges in accurately detecting road manhole covers while maintaining computational efficiency.

Accuracy and speed are two important metrics in the field of object detection for autonomous driving vehicles^2,3^. High accuracy enables object detection algorithms to precisely locate and recognize the surrounding environment on the road, while fast speed allows the model to quickly capture changes in external objects, assisting the control system in making more informed decisions to ensure vehicle safety^4^. Therefore, it is crucial to design an object detection algorithm that exhibits both high accuracy and fast speed for autonomous driving. Balancing accuracy and speed in object detection for autonomous driving is a challenge for conventional deep learning methods. In this paper, we propose a fast and accurate object detector based on an improved YOLOv5s algorithm. Our approach achieves dual enhancements in detection accuracy and speed while reducing the model's size and number of parameters. The main contributions of this paper are given below.

- A real-time manhole cover detection method based on deep learning models has been proposed: by constructing a dataset of manhole cover images and performing data augmentation and annotation, accurate detection of manhole covers on roads has been achieved. The proposed MGB-YOLO model optimizes the YOLOv5s network structure and combines MobileNet-V3, GAM, and BottleneckCSP to strike a balance between detection accuracy and model efficiency.
- The model proposed in this paper has shown performance improvement in road manhole cover detection. This method achieves an accuracy of 96.6%, with increased mAP compared to the Faster RCNN, SSD, YOLOv5s, YOLOv7 and YOLOv8s models.
- We have reduced the size and number of parameters in the model while simultaneously improving the detection speed, making it more suitable for deployment on in-vehicle embedded devices.

The rest of this paper is organized as follows. “Related work” section introduces related work on road manhole cover detection. “Methodology” section details the proposed method in this article. “Experiment” section reports experimental data, experimental procedures, experimental results, and analysis of experimental results. Finally, in “Conclusion” section, we further summarize and analyze the proposed method in this article and consider further work.

## Related work

### Object detection

With the continuous development of artificial intelligence technology and deep learning algorithms, the performance of object detectors has been significantly improved, and deep learning has gradually been applied to target detection in traffic environments. Many researchers have carried out studies on road target detection for safe traffic operation. Leveraging deep learning approaches for the analysis of traffic videos enhances roadway safety. This is achieved by distilling operationally significant safety metrics and offering comprehensive insights and directions to comprehend and interpret the traffic footage^5^. Hassaballah et al.^6^ proposed a vehicle detection and tracking approach that achieves the optimal balance between accuracy and detection speed in adverse weather conditions, applicable to various traffic environments. Bibi et al.^7^ proposed an edge AI framework based on the ResNet-18 and VGG-11 models for road anomaly detection in autonomous vehicles. The trained deep learning models were deployed at the vehicle level to automatically detect road anomalies. Güney et al.^8^ employed a real-time system based on the Faster RCNN architecture as a detection methodology. This system, utilizing a camera, is capable of detecting traffic signs and road objects within a driving environment. The developed system in this research offers ease of use for robust detection of traffic signs and objects. Hang et al.^9^ applied an improved Faster RCNN for the automatic detection of road manhole covers. The experimental results highlight the advantages of deep-learning-based object detection algorithms in the intelligent detection of pavement manhole covers. Mahaur et al.^10^ conducted a comparative analysis of the accuracy exhibited by five key image processing algorithms, namely, the Region-Based Fully Convolutional Network (R-FCN), Mask-Based Regional Convolutional Neural Network (Mask R-CNN), Single Multi-Frame Detector (SSD), RetinaNet, and You Only Look Once v4 (YOLOv4). Their conclusions emphasized that, within an identical testing milieu, YOLOv4 displayed commendable accuracy in identifying challenging road-based target objects, even under complex roadway circumstances and diverse meteorological conditions.

### Improved YOLO model

Detection methods represented by the YOLO series of object detection algorithms have exhibited high accuracy and real-time performance, leading to their widespread adoption in various scenarios. Francies et al.^11^ validated the application of the YOLO algorithm for the detection and recognition of multiple 3D objects, substantiated through experimentation on the Pascal VOC dataset. Li et al.^12^ equipped a vehicle with an intelligent traffic sign recognition system to mitigate potential safety risks engendered by human cognitive inaccuracies. They proposed a YOLO-based model specifically for traffic sign recognition. This proposed algorithm was put to the test with a dataset centered around German traffic signs, and was subsequently juxtaposed with other benchmark algorithms. Findings indicate that the algorithm not only maintains high classification precision but also considerably bolsters execution speed. Addressing the shortcomings of YOLO v3, Yang et al.^13^ implemented optimizations to the algorithmic architecture, the K-means clustering algorithm, and the loss function. These adjustments substantially enhanced the accuracy and speed of the detection framework, effectively mitigating issues related to low precision in road traffic sign recognition. Addressing the issues of low detection accuracy inherent in traditional image processing methods and poor real-time performance associated with deep learning-based methods, Huu et al.^14^ proposed a dual-lane detection algorithm based on computer vision. This involved the utilization of a pre-trained YOLO model to identify obstacles within images captured by the vehicle's front camera. Results demonstrated an improvement in average accuracy and a reduction in execution time.

In an experiment conducted by Zhu et al.^15^ the performance of the latest version of YOLOv5 was evaluated based on a traffic sign recognition dataset they created. Through a comprehensive comparison with SSD, YOLOv5 outperformed SSD in terms of mAP@0.5 across all categories. Additionally, YOLOv5 demonstrated superior recognition speed compared to SSD. In his 2022 work, Li deployed deep learning-based object detection techniques to the process of detecting objects in aerial photography of highways. Experimental evidence demonstrated that the refined YOLOv5 network is capable of more precise and efficient identification and localization of objects on aerially photographed roads^16^. Guo et al.^17^ introduced an enhanced road damage detection method based on the YOLOv5s model, employing substantial data to train the network model to detect various pavement cracks. The experiments indicate that the model's detection accuracy and inference speed can be improved by optimizing the backbone network and incorporating a Coordinate Attention module into the proposed method. Dang et al.^18^ improved the YOLOv5s model under adverse weather conditions such as low light, rainy, and foggy weather. The results indicated a significant improvement in the accuracy of the traffic sign recognition model for detecting blurred distant objects. In order to enhance the detection accuracy of small objects such as traffic signs and traffic lights under adverse weather and low-light conditions, Mahaur et al.^19^ optimized the YOLOv5 model, which significantly improved the precision and speed of small object detection.

However, YOLO still has certain limitations when dealing with small and densely packed objects, highlighting the need for further improvement and optimization. In conclusion, despite the existing research efforts in road surface cover detection, there are still unresolved issues and challenges that require urgent attention. Based on the insights gained from previous research, this paper aims to address the limitations of the YOLO algorithm and propose improvements. The study introduces a novel and efficient method for detecting manhole covers using binocular depth cameras mounted on vehicles. By incorporating MobileNet-V3, GAM, and BottleneckCSP^20^, the YOLOv5s network model is enhanced to achieve a lightweight design while maintaining high detection accuracy. Comparative evaluations with other prevalent target detection methods are conducted to validate the effectiveness of the proposed approach. This paper provides a comprehensive overview of the design and implementation process of the proposed method, accompanied by a thorough performance evaluation.

## Methodology

### Review of YOLOv5

In the selection process for target detection algorithms, speed and efficiency are paramount considerations for a plethora of object detection tasks. This is particularly relevant in in-vehicle target detection scenarios, where the ability to perform real-time object detection is crucial. Among available options, the YOLO algorithm stands out. It facilitates object detection with just a single traversal of the neural network, significantly outpacing many competing object detection algorithms^21^. Diverging from some counterparts that separate object detection and subsequent classification, YOLO accomplishes both tasks simultaneously. By treating object detection as a solitary regression problem, YOLO predicts class probabilities and bounding box coordinates in a singular step, further streamlining the process. Given identical hardware conditions, YOLOv5 demonstrates superior performance by achieving faster inference speeds and a more efficient utilization of computational resources across several commonly used datasets. The algorithm is complemented by an array of tools and features that enhance the convenience of model training and fine-tuning processes.

YOLOv5s represents the smallest model variant within the YOLOv5 algorithm series. Its objective is to maintain a certain level of performance while optimizing the speed and size of the model, making it suitable for operation on resource-constrained devices such as mobile and embedded systems. The network architecture of YOLOv5s primarily comprises three components: the backbone network, the neck network, and the detection network. The backbone network serves to extract features from images^22^. Within YOLOv5s, this backbone network consists of multiple convolutional layers, each handling images of differing granularity and generating corresponding image features. The primary role of the neck network is to integrate the various features extracted by the backbone network. By merging feature maps of different scales, the neck network is capable of generating feature maps that encapsulate rich semantic information and positional data. The detection network is responsible for the final task of object detection. It utilizes the feature maps generated by the neck network to predict the categories and locations of objects^23^.

### Enhanced backbone with MobileNet-V3

**Figure 1:**
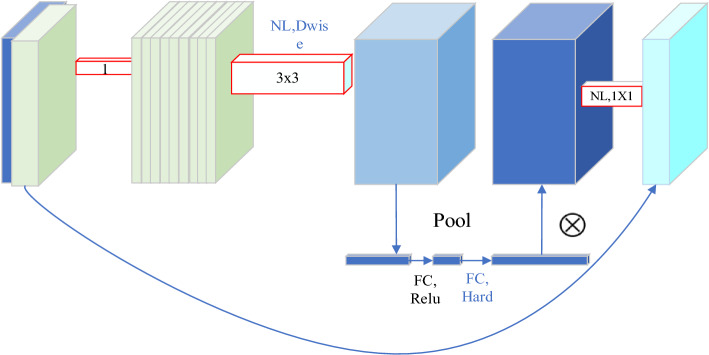
Structure of MobileNet-V3 model.

MobileNet-v3^24,25^ represents a lightweight and efficient neural network architecture, delivering exceptional performance in tasks demanding limited computational resources without substantially compromising accuracy. MobileNet-V3 is designed to offer efficient performance when deployed on mobile and embedded devices. This objective is achieved by reducing model complexity and size, and by making hardware-aware optimizations to enhance speed and energy efficiency^26^. Concurrently, MobileNet-V3 also incorporates a novel non-linear activation function hard-swish, along with adaptability modifications for small models, including larger input sizes and a higher dimensionality of the final layer. The structure of MobileNet-V3 is illustrated in Fig. 1.

MobileNet-v3 is a convolutional neural network architecture, representing the third iteration of the MobileNet series, with significant improvements in accuracy and efficiency over its predecessors. By employing lightweight building blocks and incorporating advanced features such as squeeze-and-excitation modules, MobileNet-v3 achieves a balanced trade-off between accuracy and efficiency. To ensure a lightweight model, considerations were made for its use in automotive mobile devices and embedded vision applications.

In the model backbone, the first bottleneck layer, C3, is replaced with MobileNet-v3. Before the second C3 of the original backbone, a MobileNet-v3 module is added, while the third C3 module is eliminated. To enhance the model’s ability to capture global contextual information and improve object detection performance, the self-attention mechanism is introduced. The GAM module substitutes the final C3 of the YOLOv5s backbone network.

### Global attention mechanism

With the advancement of deep neural networks, the attention mechanism has been extensively utilized across various application domains^27^. The GAM represents a widely implemented attention mechanism across various deep learning models, permitting the model to consider all positions within the input sequence while generating new hidden states. This mechanism enables the model to better understand and process the contextual information within images. It assists the model in comprehending the relative positioning of objects and in managing complex backgrounds more efficiently. Within object detection tasks, this translates into more precise identification of small or densely placed objects, as well as improved handling of obstructions and overlaps^28^.

**Figure 2:**
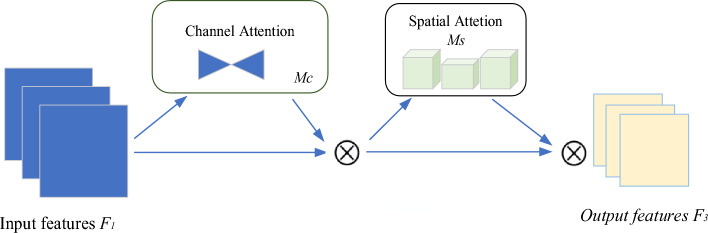
Structure of the GAM model.

The process of GAM is illustrated in Fig. 2, and it is mathematically represented by Eqs. (1) and (2). The input feature mapping, denoted as $F_{1}$, the intermediate state, denoted as $F_{2}$, and the output, denoted as $F_{3}$, are defined as follows:

1 $$F_{2} = M_{c} (F_{1} ) \otimes F_{1}$$

2 $$F_{3} = M_{s} (F_{2} ) \otimes F_{2}$$

where $M_{c}$ and $M_{s}$ represent channel and spatial attention maps, and ⊗  denotes element multiplication.

### Improvement of the neck network

**Figure 3:**
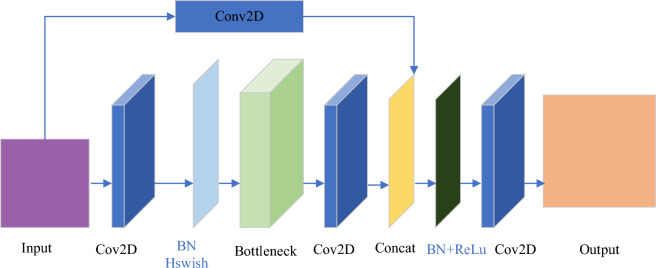
Structure of BottleneckCSP model.

BottleneckCSP represents an efficient design pattern for deep neural networks, integrating two complementary approaches to facilitate high-performing information processing within the network architecture. This structure exhibits considerable capacity for reducing parameter count and alleviating computational load, while simultaneously preserving efficient information flow. As a result, models employing BottleneckCSP tend to maintain high levels of precision, whilst demonstrating rapid inference speed and a reduced memory footprint^29^. When incorporated with YOLO, BottleneckCSP offers substantial advantages in terms of effective feature extraction. Models such as YOLOv4 utilize BottleneckCSP to architect their backbone network, enabling rapid and efficient extraction of salient features from input imagery, thereby realizing robust performance in object detection tasks^30^. The structure of BottleneckCSP is illustrated in Fig. 3.

The principal advantage of the BottleneckCSP architecture lies in its ability to reduce computational complexity while maintaining a rich flow of information. This technique enhances the reusability of features, consequently improving the model's representational capacity. In an effort to enhance the detection performance of the YOLOv5s model, diminish computational costs, and preserve the model's capability to handle complex tasks while achieving lightweight efficiency, this paper presents an approach in which the C3 module in the Neck part of the YOLOv5s network model is replaced with BottleneckCSP.

### Loss function

The YOLOv5s network's loss function, denoted as $L_{object}$, is fundamentally composed of three principal components: the bounding box regression loss $\left( {L_{loc} } \right)$, the confidence loss $\left( {L_{conf} } \right)$, and the target classification loss $\left( {L_{class} } \right)$,the loss function is calculated as follows:

3 $$L_{object} = L_{loc} + L_{conf} + L_{class}$$

4 $$L_{loc} = 1 - GIoU$$

5 $$L_{conf} = - \sum\limits_{i = 0}^{K \times K} {I_{ij}^{obj} \left[ {\hat{C}_{i}^{j} \log C_{i}^{j} + \left( {1 - \hat{C}_{i}^{j} } \right)\log \left( {1 - C_{i}^{j} } \right)} \right]} - \lambda_{noobj} \sum\limits_{i = 0}^{K \times K} {\sum\limits_{j = 0}^{M} {I_{ij}^{noobj} \left[ {\hat{C}_{i}^{j} \log C_{i}^{j} + \left( {1 - \hat{C}_{i}^{j} } \right)\log \left( {1 - C_{i}^{j} } \right)} \right]} }$$

6 $$L_{class} = - \sum\limits_{i = 0}^{K \times K} {I_{ij}^{obj} } \sum\limits_{c \in classes} {\left[ {\hat{P}_{i}^{j} \log P_{i}^{j} + \left( {1 - \hat{P}_{i}^{j} } \right) + \log \left( {1 - P_{i}^{j} } \right)} \right]}$$

where *K* represents the feature map output by the network being divided into *K* × *K* grids, *M* represents the number of anchor boxes corresponding to each grid, $I_{ij}^{obj}$ represents the anchor box with a target, $I_{ij}^{noobj}$ represents the anchor box without a target, and $\lambda_{noobj}$ represents the confidence loss weight coefficient for the anchor boxes without a target.

The confidence loss within the YOLOv5s loss function serves as a crucial measure of the model's ability to predict the presence of an object within a given anchor box. Notably, a higher value of parameter $\lambda_{noobj}$ in the loss function indicates that the model assigns greater weight to anchor boxes without a target, consequently intensifying its focus on false positives. However, the detection of small targets, exemplified by road manhole covers, presents inherent challenges due to their limited size and distinct features. When parameter $\lambda_{noobj}$ is set to a higher value, the model becomes more sensitive to false positives, potentially leading to erroneous detections of small objects, including false positives associated with manhole covers. To address this concern and enhance the model's performance in detecting small targets, the paper suggests reducing the value of parameter $\lambda_{noobj}$. By doing so, the model allocates lesser significance to anchor boxes without targets, effectively mitigating false positives and significantly improving the accuracy of small target detection.

**Figure 4:**
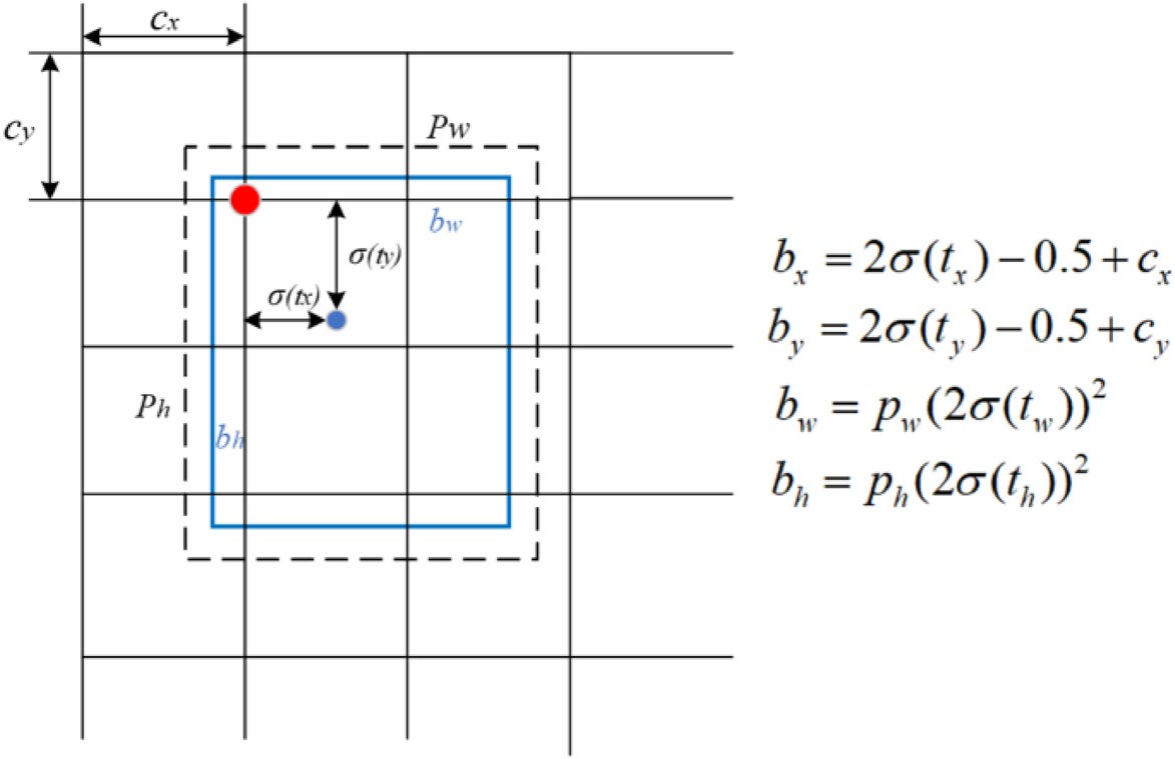
Schematic diagram of target box regression.

In the YOLOv5s model, CIoU (Complete Intersection over Union) considers the distance between the center points when calculating the intersection over union. Therefore, it provides a better description of the position relationship of the predicted boxes compared to GIoU (Generalized Intersection over Union). CIoU takes into account the position relationship of the predicted boxes more comprehensively, especially when the target is far from the detector and relatively small, enabling better differentiation between different positions of the predicted boxes. Consequently, this paper selects CIoU as the loss function for target regression. The formula for CIoU is as follows:

7 $$CIoU = IoU - \frac{{\rho^{2} (b,b^{gt} )}}{{c^{2} }} - \alpha \nu$$

8 $$\alpha = \frac{\nu }{{\left( {1 - IoU} \right) + \nu }}$$

9 $$\nu = \frac{4}{{\pi^{2} }}\left( {\arctan \frac{{w^{gt} }}{{h^{gt} }} - \arctan \frac{w}{h}} \right)^{2}$$

where $\alpha$ is an equilibrium parameter that does not participate in gradient calculation, and $\nu$ is a parameter used to measure the consistency of aspect ratio. The schematic diagram of target box regression is illustrated in Fig. 4.

Where $t_{x}$, $t_{y}$, $t_{w}$, and $t_{h}$ represent the offsets, *σ* represents the Sigmoid activation function, which is used to map the network predicted values of $t_{x}$, $t_{y}$, $t_{w}$, and $t_{h}$ to the [0,1] interval.$c_{x}$ and $c_{y}$ represent the offsets in the grid cells relative to the upper left corner of the image.$p_{w}$ and $p_{h}$ respectively represent the width and height of the prior box. $b_{x}$, $b_{y}$, $b_{w}$, and $b_{h}$ represent the central coordinates of the predicted target box.

### The proposal of the MGB-YOLO model

In the realm of deep learning models, there is a correlation between network depth within a certain range and the complexity of its composition. Generally, as the network depth increases, the accuracy of target recognition is likely to improve. However, this comes at the cost of significant computational resources, requiring more floating-point operations, and resulting in large memory footprints for the model weight files. Consequently, this escalates the hardware requirements for deployment, encompassing CPU computational capabilities, operating memory, and storage space^31^. Given these considerations, and bearing in mind that the manhole cover model is primarily deployed on embedded controllers and mobile hardware platforms, there is a marked preference towards lightweight models.

**Figure 5:**
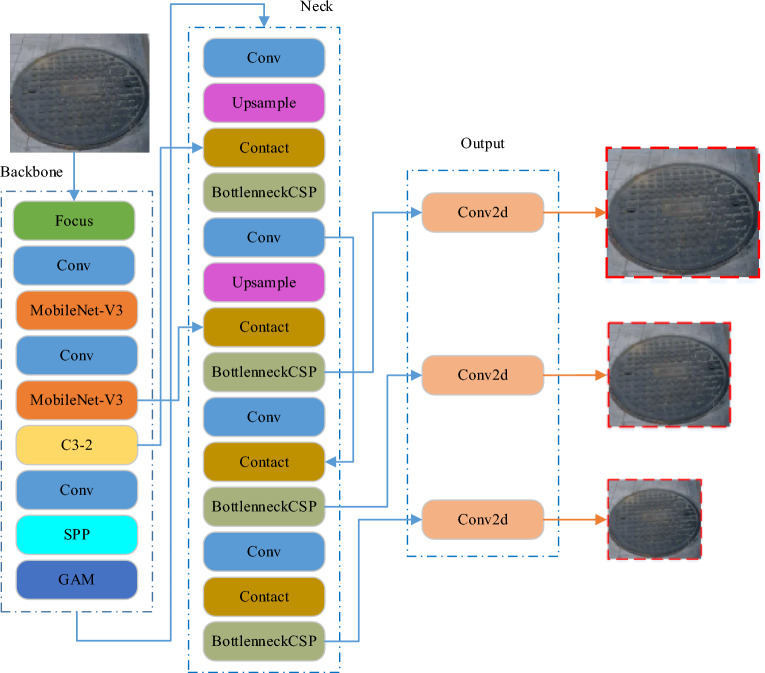
Structure of MGB-YOLO (Ours) model.

Within the YOLOv5 series, there exist four fundamental models: YOLOv5x, YOLOv5l, YOLOv5m, and YOLOv5s. YOLOv5s stands out for its smaller memory footprint and faster detection speed, all the while maintaining a high degree of accuracy. For rapid and accurate detection of manhole cover targets on road surfaces, we propose a new network model based on YOLOv5s. This model combines YOLOv5s with MobileNet-V3, GAM, and BottleneckCSP. MobileNet-V3 focuses on efficient performance and performs excellently on resource-constrained devices. With the introduction of the Global Attention Mechanism, the model achieves higher accuracy in tasks that rely on extensive context and global element relationships. BottleneckCSP introduces cross-stage connections, facilitating the exchange of information between different stages of the network, enhancing the network’s ability to capture features at multiple scales, optimizing computation and parameter efficiency, and resulting in faster training and inference speeds. We call this network model MGB-YOLO. The structure of the MGB-YOLO model network is illustrated in Fig. 5.

## Experiment

### Image date acquisition

**Figure 6:**
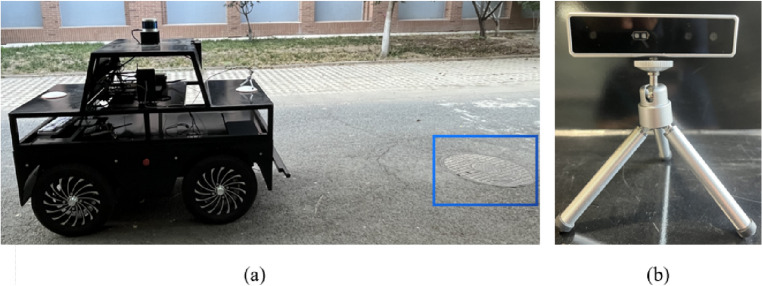
The equipment used to collect data. (**a**) Mobile robot. (**b**) Binocular stereo depth camera.

At present, no image dataset specifically catered to manhole covers on road surfaces exists. To address this gap, our study curated a unique selection of target images. These images were captured on a campus road at a university located in the Haihe Education Park, Tianjin, China. Our collection comprised a total of 1000 target images, representing both standard, undamaged manhole covers, as well as those exhibiting varying degrees of damage. This balanced dataset aids in fostering a comprehensive understanding of both normal and non-normal conditions. The data collection equipment is shown in Fig. 6.

**Figure 7:**
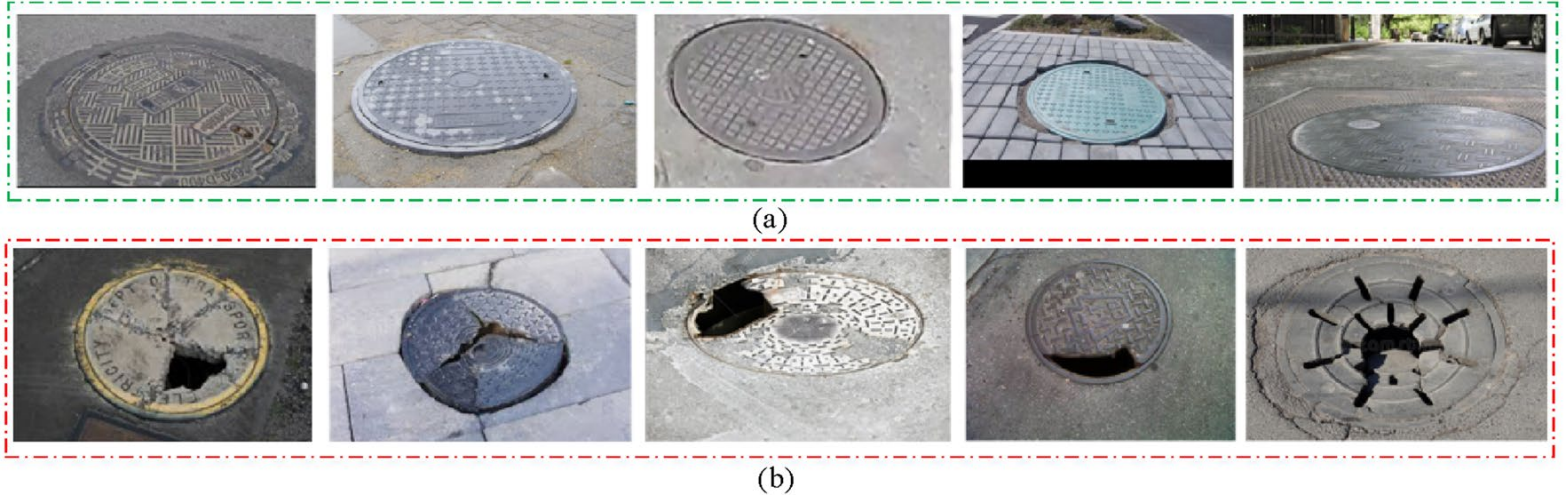
Selected images in the dataset. (**a**) Qualified manhole covers; (**b**) unqualified manhole covers.

Figure 7 provides a visual representation of a subset from our dataset, showcasing both qualified (standard, undamaged) and unqualified (damaged) manhole covers.

### Image data augmentation

**Figure 8:**
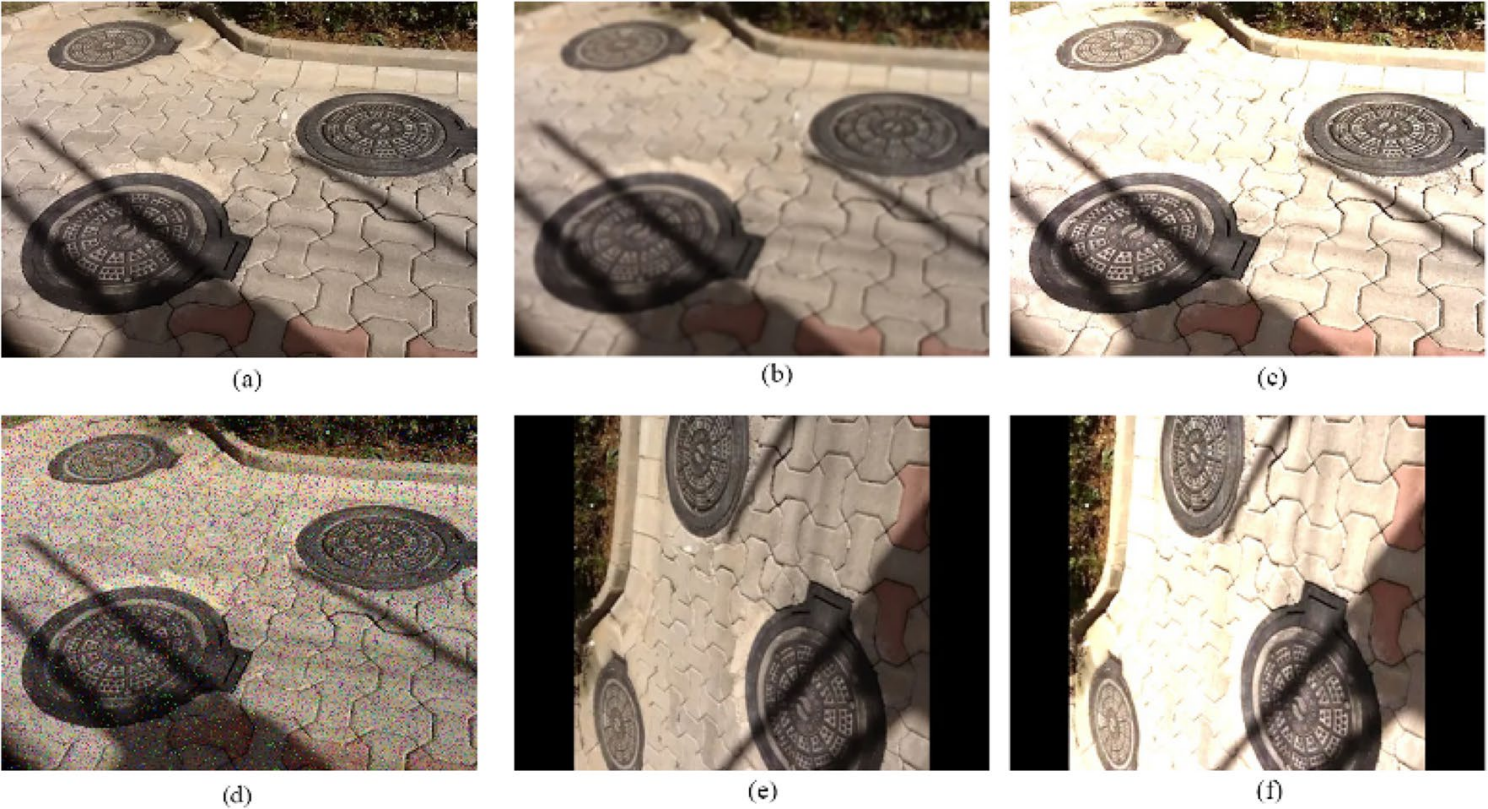
Example image data amplification effect (**a**) origin picture; (**b**) gaussian blur; (**c**) brighter; (**d**) noise; (**e**) R90; (**f**) R90 and brighter.

In the training of deep learning models, an insufficient amount of data may give rise to issues such as overfitting, suboptimal model performance, and inadequate generalization. To mitigate these problems, it is essential to augment the dataset, thereby increasing the quantity and diversity of the samples. In our study, we employed five data augmentation techniques, namely: original images, Gaussian blur, brightness adjustment, noise addition, and rotation by 90° (both solely and in combination with brightness adjustment). These techniques are illustrated in Fig. 8. By applying these methods, we successfully expanded the sample size from 1000 to 6000, significantly enhancing the robustness and generalizability of our model.

### Experimental environment

The experimental procedure was executed on a Windows 10 operating system, powered by an Intel(R) Core(TM) i7-13700KF CPU with a clock speed of 3.40 GHz and a Nvidia GeForce RTX 4080 graphics card. The deep learning undertakings leveraged the PyTorch1.8.1 framework, with PyCharm serving as the primary development environment, and Python 3.9 as the designated programming language. All algorithms utilized in comparative analysis were operationally consistent, being run within the same computational setting. During training, manual selection and normalization were employed to filter all existing images. The image size was standardized to 640 × 640 × 3, and the format was unified to .jpg. the batch size is 32, and the learning rate is set at 0.001, the number of training epochs is 200.

### Evaluation criteria

In this study, we employ a suite of four key metrics to appraise the performance of the detection model, specifically, Average Precision (AP), mAP, Precision, and Recall. The evaluation process involves a comprehensive consideration of four distinct predictive scenarios: True Positives (*TP*) represent correct predictions of positive samples; True Negatives (*TN*) illustrate correct predictions of negative samples; False Positives (*FP*) indicate incorrect predictions of negative samples; and False Negatives (*FN*) denote incorrect predictions of positive samples. By analyzing the proportions of these four scenarios, we calculate the values of Precision and Recall, which subsequently aid in the determination of other performance indicators.

Where *P* denotes the Precision. Pertaining to the final prediction outcome, Precision is a measure of the number of positively predicted samples that were indeed positive, as expressed in Eq. (10):

10 $$P = \frac{TP}{{TP + FP}} \times 100\%$$

where R denotes the Recall rate. In the context of the original dataset, Recall represents the proportion of positive cases that the model accurately predicted, as defined by Eq. (11):

11 $$R = \frac{TP}{{TP + FN}} \times 100\%$$

In the actual experiments, the Recall and Precision were consistently high. To evaluate the performance of the algorithmic network, a parameter called mAP was introduced, which combines Recall and Precision. mAP is specifically designed for multitarget detection and can be represented as Eq. (12)

12 $$mAP = \frac{1}{c}\sum\limits_{k = 1}^{N} {P(k)\Delta R(k)}$$

In Eq. (12), *N* represents the total number of samples present in the validation set.$P\left( k \right)$ indicates the precision achieved when k targets are simultaneously detected, while $\Delta R\left( k \right)$ represents the change in recall as the number of detected samples transitions from *k-1* to *k*. Additionally, *C* denotes the number of classes considered by the model. In this study, the sole requirement is to identify the qualified and unqualified manhole covers, thus $c = 2$.

### Experimental results

**Figure 9:**
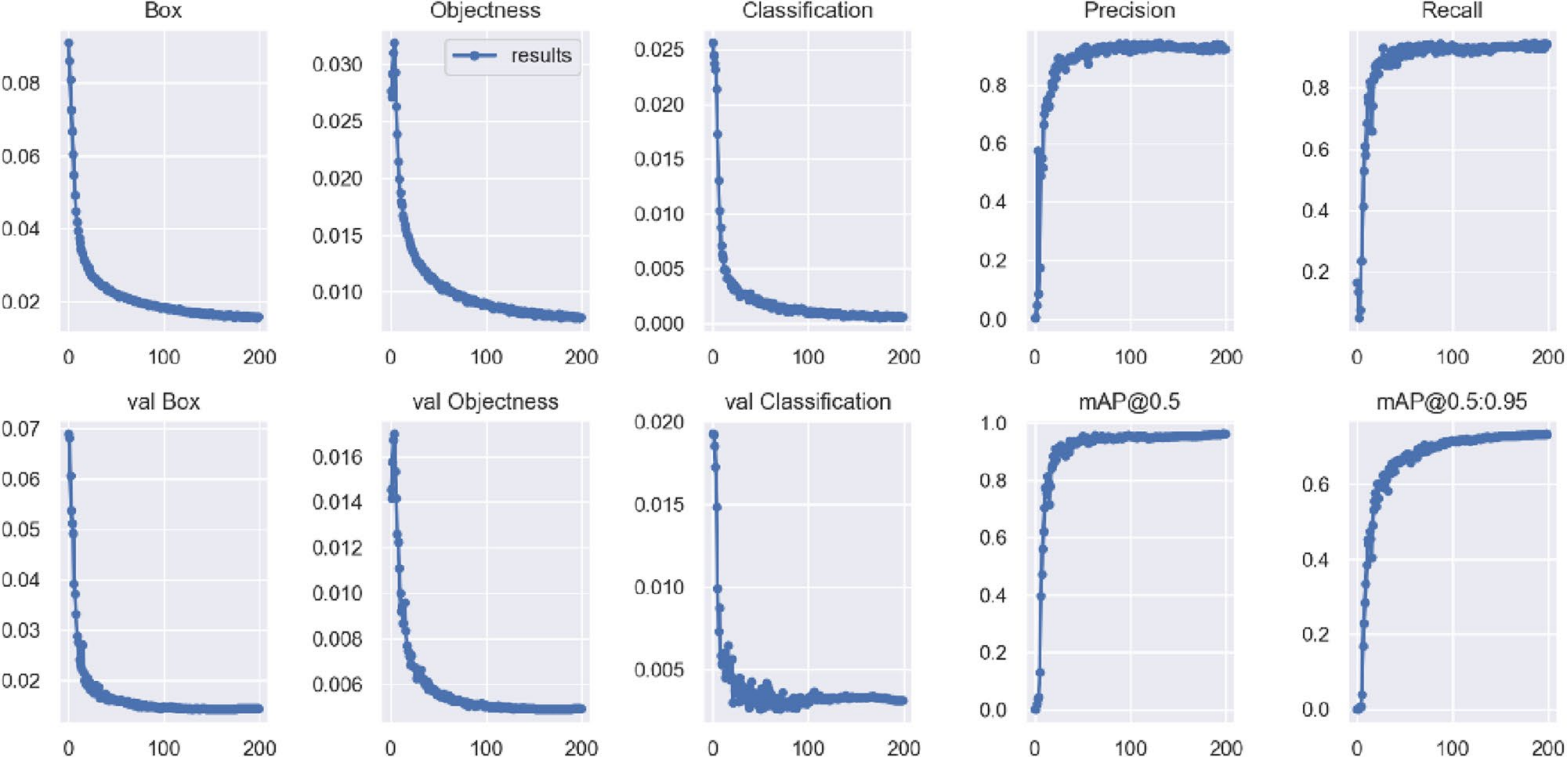
All specific evaluation metrics of MGB-YOLO in our dataset.

To assess the training process of the model and the effectiveness of object detection, the experiment uses box loss, objectness loss, class loss, precision, recall, mAP@0.5, and mAP@0.5:0.95 from the training and testing datasets as primary parameters to gauge the degree of convergence. For the six loss functions, including (val) box loss, (val) objectness loss, and (val) class loss, a smaller parameter value indicates better training performance. Figure 9 illustrates the relationships between the six loss functions and elapsed time, as well as accuracy, recall, and mAP values over time. During the first 100 epochs of network training, the loss values decrease rapidly, and they tend to stabilize after 200 epochs.

**Figure 10:**
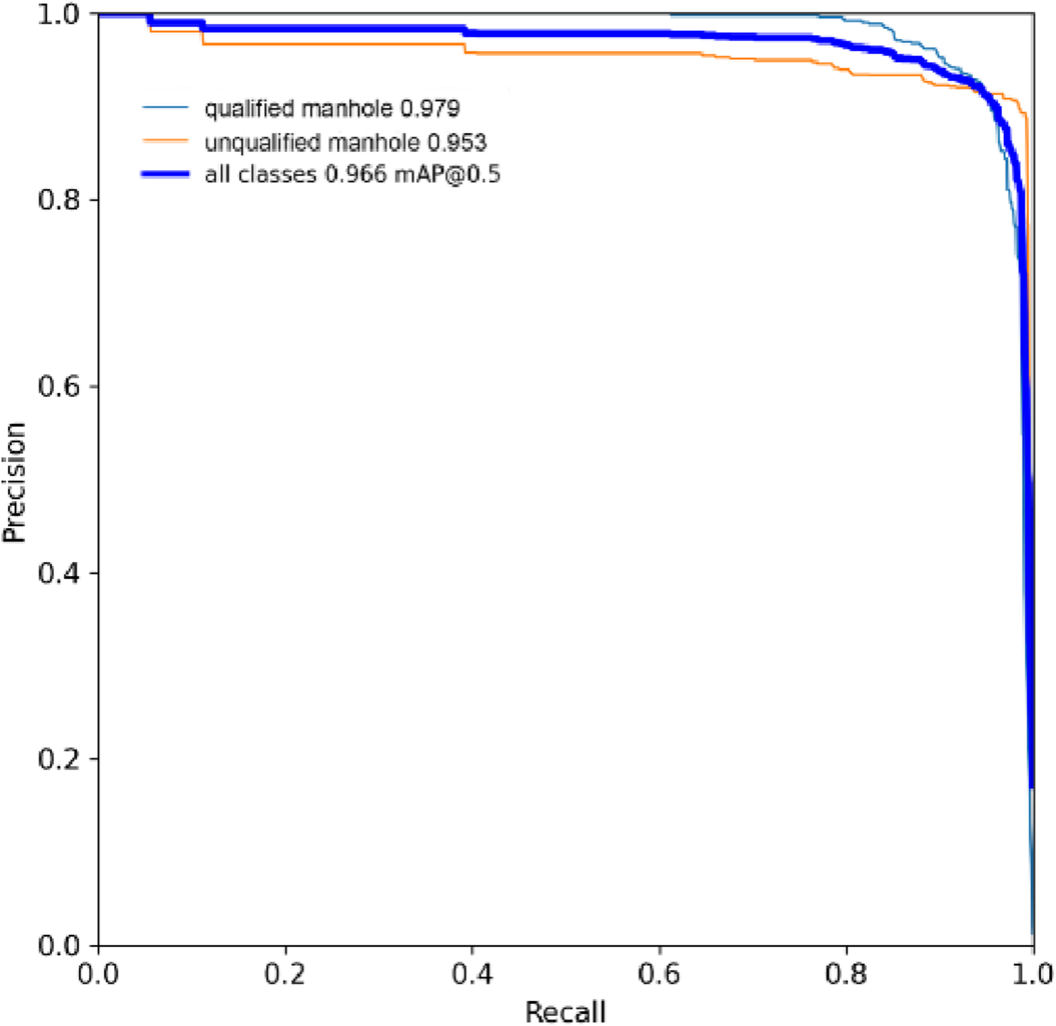
P-R curve of MGB-YOLO.

The dataset of qualified and unqualified manhole covers was used to train the MGB-YOLO network, with the precision–recall (P–R) curve shown in Fig. 10. As can be inferred from the data in Fig. 10, the overall testing performance is relatively satisfactory. The mAP of the qualified manhole covers is 97.9%, and that of the unqualified manhole covers is 95.3%, with a combined classification mAP of 96.6%. The reason for this curve effect is that the number of qualified manhole covers is greater than that of unqualified ones, thereby yielding better testing results.

### Experimental comparisons of different models

In order to validate the performance of the proposed model, the MGB-YOLO, trained with a manhole cover dataset, was compared with the YOLO v5s, SSD, Faster RCNN, YOLOv7, and YOLOv8s models. Through this approach, the superior performance of the proposed model was demonstrated. The mAP, model size, and detection speed of the MGB-YOLO compared with the YOLO v5s, SSD, Faster RCNN with Resnet 50, YOLOv7, and YOLOv8s are presented in Table 1. Due to the SSD architecture based on VGG16 typically not automatically adapting to various input image sizes but instead using a fixed input image size, experiments were conducted with a resolution of 512 × 512 pixels, while other algorithms employed an input image size of 640 × 640 pixels.

**Table 1:** mAP results, model size, detection speed, parameters and floating-point numbers for four models.

Model	mAP (%)	Model size (MB)	FPS	Parameter (M)	FLOPs (GFLOPS)
MGB-YOLO	96.6	14.03	59	7.3	13.5
YOLOv5s	95.9	14.51	55	7.5	16.5
SSD	89.7	186.61	67	13.8	15.3
Faster RCNN	81.0	315	40	41.35	41.35
YOLOv7	96.1	72.984	6	37.20	105.1
YOLOv8s	96.1	22.946	50	11.12	28.4

**Figure 11:**
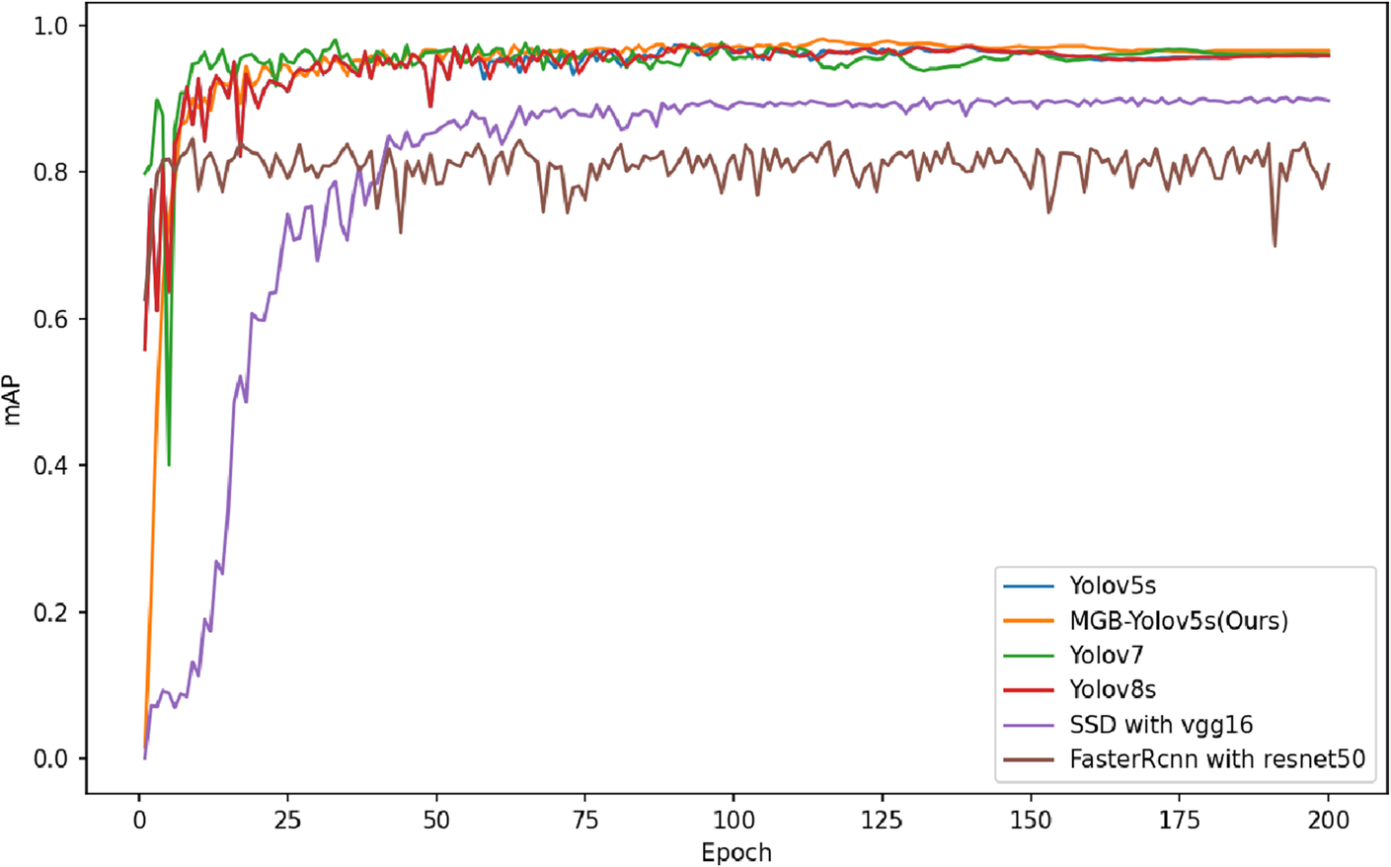
mAP value as a function of gradually increasing epoch.

The same parameters were set for the experiments, along with identical hardware and software environments. The training was conducted for a total of 200 iterations. The mAP curves of the four models are displayed in Fig. 11. As depicted in Table 1 and Fig. 11, which elucidate the mAP comparison outcomes among diverse models, it is observable that the MGB-YOLO model's mAP transcends that of the other models. The detection precision that it attains surpasses the YOLOv5s model by an increment of 0.7%, the SSD model by 6.9%, and notably outstrips the Faster RCNN model by a significant 15.6% margin. Of all the models under scrutiny, the MGB-YOLO model demonstrates the smallest footprint, with a size merely measuring 14.03 MB. The quantity of parameters and the floating point operations budget for the MGB-YOLO model are estimated to be 7.3 million and 13.5 FLOPs. Compared to the state-of-the-art YOLOv7 and YOLOv8s algorithms, the mAP of the MGB-YOLO model only exhibits a modest improvement of 0.5%. However, the model’s storage footprint is significantly smaller than YOLOv7 by 58.954 MB and YOLOv8s by 8.916 MB. Taking into consideration the overall model parameter quantity, the MGB-YOLO model is better suited for deployment on in-vehicle embedded devices. In order to achieve more stable and reliable results, we ran the MGB-YOLO algorithm five times with the same parameters. The mAP values obtained were 96.6%, 96.9%, 96.4%, 96.1%, and 96.6%, with an average mAP of 96.6%.

**Figure 12:**
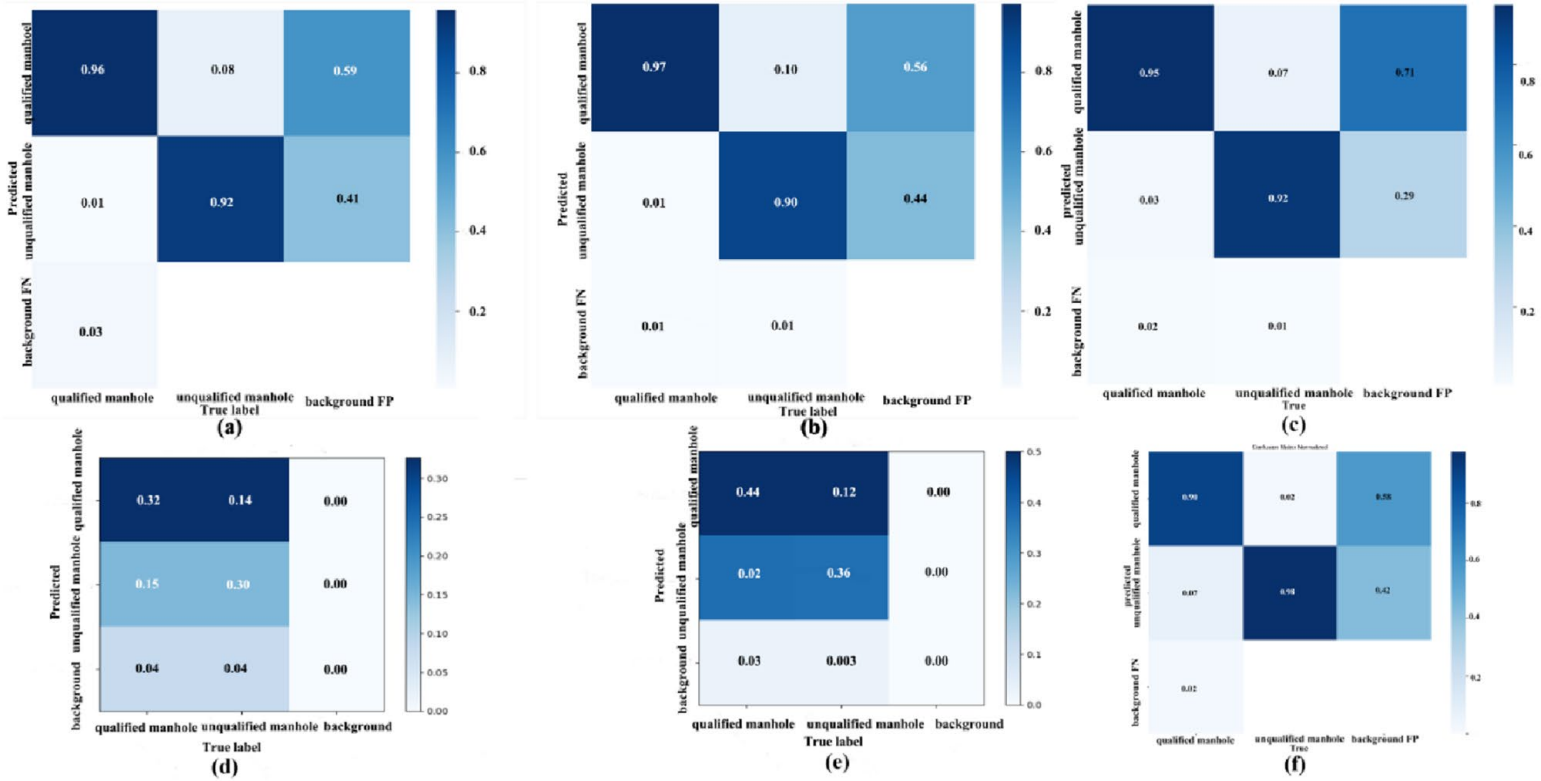
The confusion matrix of the comparison of the different defect detection algorithms (**a**) MGB-YOLO; (**b**) YOLOv5S; (**c**) Faster RCNN; (**d**) SSD; (**e**) YOLOv7; (**f**) YOLOv8s.

In Table 1, we summarize the performance of four deep learning network models in the test dataset, with their respective confusion matrices presented in Fig. 12. From the confusion matrices, we observed that MGB-YOLO leads, even significantly outperforms, the Faster RCNN and SSD models in terms of correctly classifying both qualified and unqualified manhole covers. In the correct classification of qualified manhole covers, MGB-YOLO is on par with YOLOv5s. In the correct classification of unqualified manhole covers, however, YOLOv5s has a lower accuracy rate than MGB-YOLO. Overall, within the confusion matrices, MGB-YOLO's classification performance is superior to the other three models. However, for background detection, the classification accuracy of all four models is relatively low. This phenomenon can be attributed to the diverse shapes in the background, which poses certain difficulties for the feature extraction of target detection models in deep network learning models. This is also a common challenge in the field of small target detection.

**Figure 13:**
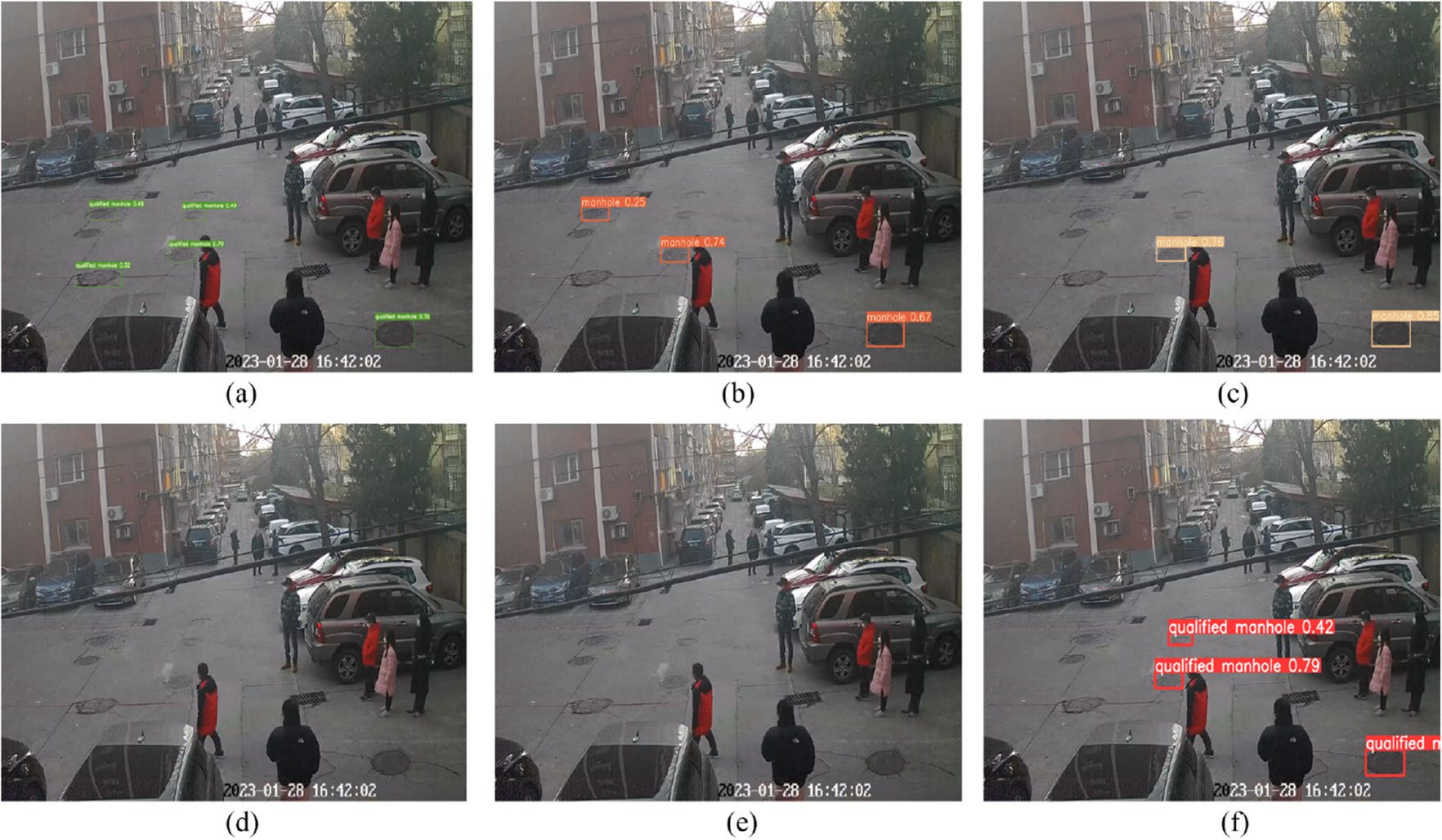
Detection effects of different models: (**a**) MGB-YOLO; (**b**) YOLOv5S; (**c**) Faster RCNN; (**d**) SSD; (**e**) YOLOv7; (**f**) YOLOv8s.

Figure 13 presents the practical detection results of the MGB-YOLO algorithm and other algorithms under the same traffic conditions. The MGB-YOLO algorithm accurately identifies 5 manhole covers, achieving the highest correct recognition count and demonstrating superior universality and applicability. Compared to other algorithms, the MGB-YOLO algorithm exhibits higher accuracy in detecting the same objects, particularly during the process of detecting small targets.

**Figure 14:**
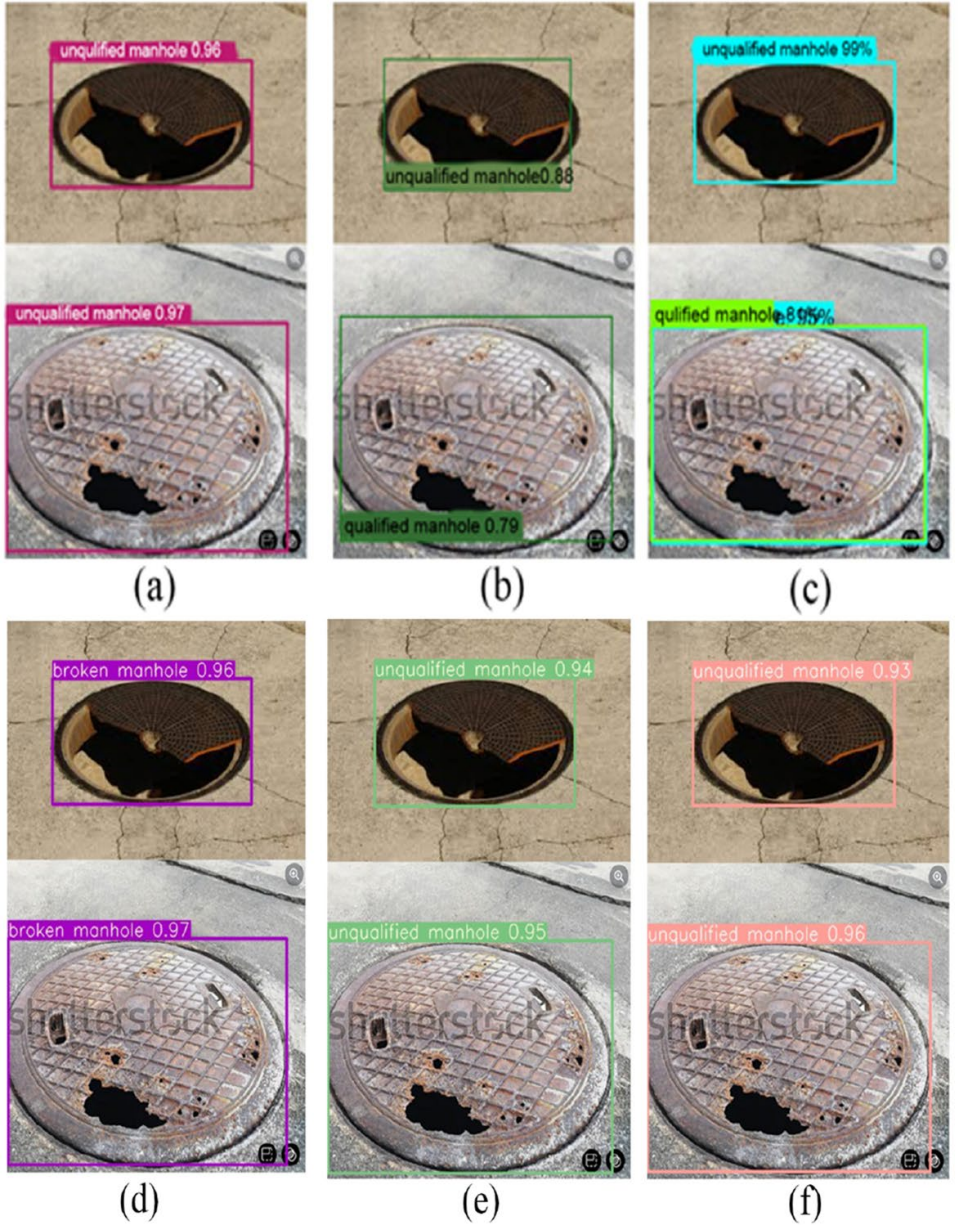
Detection effects for unqualified manhole: (**a**) MGB-YOLO; (**b**) SSD; (**c**) Faster RCNN; (**d**) YOLOv5s; (**e**) YOLOv7; (**f**) YOLOv8s.

Figure 14 presents the real-world detection outcomes of the MGB-YOLO algorithm in comparison to Faster RCNN and SSD for detecting unqualified manhole covers. The MGB-YOLO algorithm effectively diminishes the miss detection and error rates. Conversely, the SSD algorithm erroneously classifies unqualified manhole covers as qualified. While Faster RCNN inconsistently categorizes unqualified manhole covers as both qualified and unqualified. In Fig. 14c, the text in the chart is occluded, which is attributed to the Faster RCNN algorithm mistakenly recognizing both qualified and unqualified well covers simultaneously. This indicates a potential misjudgment of the Faster RCNN algorithm in the recognition process on this particular dataset.

The recognition experimental results presented in Figs. 13 and 14 indicate that the MGB-YOLO algorithm effectively reduces both omission and error rates, and it provides a higher level of accuracy for small targets. These results demonstrate the effectiveness and superiority of the proposed algorithm.

## Conclusion

In this paper, we present an enhanced version of the YOLO v5s model, termed MGB-YOLO, designed specifically for road manhole cover detection. To strike an optimal balance between detection precision and computational speed, we have incorporated the MobileNet-V3, GAM, and BottleneckCSP into the network architecture. To validate the efficacy of our proposed approach, we juxtaposed it with several state-of-the-art detection methods. The experimental outcomes reveal that, relative to the YOLO v5s model, the MGB-YOLO model achieves considerable enhancements in detection speed while sustaining a comparable level of accuracy. Furthermore, when juxtaposed with SSD, Faster RCNN, YOLOv7 and YOLOv8s, it showcases evident superiority concerning recognition precision and model light-weights. While MGB-YOLO is currently applicable for road manhole cover detection, there is room for improvement in terms of aligning its performance with real-time detection standards. Future endeavors will focus on refining the current model to ensure that manhole cover detection in videos fulfills real-time requirements. Plans to deploy this model on vehicular embedded devices are underway to provide superior portability in real-world applications. Additionally, we intend to fine-tune our data augmentation strategies to further enhance the detection accuracy.

## Data Availability

The data provided in this study can be obtained from the corresponding author D.P.
